# Synergistic Effect of Chopped Fibers and Basalt Fiber Textile on Concrete Flexural Behavior

**DOI:** 10.3390/ma19204323

**Published:** 2026-10-14

**Authors:** Chaobin Hu, Liping Li, Hongbin Jiang, Changrong Fu, Pan Zhou, Yao Hu

**Affiliations:** 1 School of Engineering, Lishui University, Lishui 323000, China; 2 State Key Laboratory of Safety and Resilience of Civil Engineering in Mountain Area, Nanchang 330013, China; 3 School of Civil Engineering, Chongqing University, Chongqing 400045, China

**Keywords:** textile reinforced concrete, basalt fiber textile, hybrid effect, flexural toughness

## Abstract

To reveal the synergistic effect of chopped fibers and basalt fiber textile in different failure stages of concrete, this study investigated the hybrid effects of chopped basalt fibers, polypropylene fibers, and end-hooked steel fibers and basalt fiber textile on concrete at the stages of initial cracking, crack propagation, and post-peak softening. The four-point bending test results show that the basalt fiber textile has limited effect on the initial crack strength but can increase the ultimate load by 19.98% and the ultimate deflection by 175.94% compared with plain concrete by inhibiting macroscopic crack propagation. There is a complex synergistic effect between chopped fibers and fiber textile. Chopped fibers can compensate for the insufficient reinforcement of the textile in the initial cracking stage, while the textile dominates the toughness maintenance in the later stage of crack development. The hybrid system of chopped fibers and textile can enhance the flexural bearing capacity of textile-reinforced concrete. The mixture of polypropylene fibers or basalt fibers with fiber textile exhibits a post-peak embrittlement trend, while the ternary system of basalt fibers, end-hooked steel fibers, and fiber textile can achieve synergistic optimization of strength and toughness. Its peak load, bending toughness index of I20, and peak load deflection increased by 44.30%, 50.71%, and 13.76%, respectively, compared with the control group. This study provides theoretical support for the material design of high-performance basalt fiber textile-reinforced concrete and its application in thin-walled, prefabricated, and seismic strengthening engineering.

## 1. Introduction

Concrete is widely used in various engineering fields such as construction and transportation due to its excellent compressive strength, durability, and cost-effectiveness. However, its inherent defects such as low tensile strength, high brittleness, and susceptibility to cracking greatly limit its application. Although the traditional reinforcement with steel bars can effectively improve these problems, the following problems cannot be ignored: increased component weight, excessive protective layers, and the susceptibility of steel bars to corrosion. To overcome the above drawbacks, textile-reinforced concrete (TRC) emerged as a new composite material. TRC uses corrosion-resistant fiber woven mesh to replace or partially replace steel bars [1], endowing concrete components with characteristics such as light weight [2], high strength [3], and durability [4].

Many meaningful explorations have been conducted on the reinforcing effect of TRC. Ding et al. investigated the strengthening effect of glass fiber textile on the bearing capacity of concrete slabs, and the results showed that glass fiber textile can increase the ultimate bearing capacity of concrete slabs by 59% [5]. Li et al. incorporated basalt/glass fiber hybrid textile and carbon/glass fiber hybrid textile to engineer cement-based composite materials (ECCs) and found that the hybrid textiles can improve the bearing capacity and ultimate tensile strain of ECCs [6]. Deng et al. improved the ultimate bearing capacity and energy absorption value of two-way concrete slabs using glass fiber textile [7]. These studies indicate that TRC has broad engineering application prospects.

Among various fiber textiles, basalt fiber textile is regarded as one of the ideal choices for preparing TRC components due to its comprehensive advantages such as wide availability of raw materials, low cost, natural high temperature resistance, corrosion resistance, high tensile strength, and good bonding performance with cement matrices [8,9]. In practical applications, the reinforcement effect of basalt fiber textile may be affected by factors such as the number of layers [10], interface slip [11,12], and uneven stress distribution [13], leading to large differences in the textile reinforcement effect. Du et al. found that only when the basalt fiber textile reaches three layers or more does it exhibit significant tensile strengthening potential [14]. Zhang et al. improved the tensile strength of concrete specimens by 85.5% using a three-layer basalt fiber textile [15]. Du et al. used a five-layer basalt fiber textile to increase the flexural strength and toughness of concrete by about 296% and 241%, respectively [16]. Zhang et al. utilized an epoxy resin-impregnated three-layer basalt fiber textile to achieve an ultimate strength of 1.85 times that of the control sample for the composite material [17].

However, increasing the number of textile layers poses significant obstacles to engineering applications. Scholars have attempted to incorporate chopped fibers into the TRC matrix to improve the reinforcement efficiency of basalt fiber textile and avoid the need for a relatively high number of layers. For example, Li and Ding achieved a 2.66-fold increase in the bearing capacity of TRC plates by mixing steel fibers with one layer of basalt fiber textile [13]. Ding et al. hybridized macro-polypropylene fibers with one layer of basalt fiber textile, achieving a reinforcing effect equivalent to that of concrete with three layers of basalt fiber textile [18]. These preliminary works reveal a promising direction: utilizing the hybrid effect of chopped fibers and fiber textile has more engineering application value as a solution to improve the reinforcement effect of TRC.

Existing hybrid studies mostly focus on the reinforcement effect of different dosages of chopped fibers and different layers of fiber textile or only use peak load as an evaluation index. They have not yet systematically revealed the synergistic effect of chopped fibers and basalt textile in different failure stages such as TRC initial cracking, crack propagation, and post-peak softening. Different types of chopped fibers (e.g., basalt fiber, polypropylene fiber, steel fiber) have significant differences in elastic modulus, tensile strength, and geometric characteristics. The synergistic mechanism and multiphase hybrid effect between them and basalt fiber textile remain unclear, especially the lack of in-depth research on the synergistic effects between textiles and combinations of chopped fibers with different elastic moduli or different geometric scales.

Therefore, the aim of this study is to systematically explore the single and mixed mixing effects of different types of chopped fibers (basalt fiber, polypropylene fiber, end-hooked steel fiber) in combination with basalt fiber textile. By designing a series of four-point bending tests of concrete prismatic specimens, we analyze the key performance indicators such as initial crack load, ultimate load, and load deflection curves, with the aim of clarifying the reinforcement and toughening effects of different fiber combinations, and identify the hybrid fiber combinations with the optimal synergistic effect. It provides experimental basis and theoretical reference for improving the reinforcement effect of basalt fiber textile and optimizing the design and application of high-performance TRC materials.

## 2. Methodology

### 2.1. Materials

The materials used in this study are as follows: Ordinary Portland cement (P·O 32.5) was selected as the cementitious material, with 28 d of compressive strength being ≥32.5 Mpa and specific surface area being ≥300 m^2^/kg to meet the early hydration demand. The fine aggregate was natural river sand (apparent density 2650 kg/m^3^), the particle size range was 0.3~0.5 mm, the fineness modulus was 2.7 ± 0.2, and the mud content after washing was less than 3% to ensure the quality of the interfacial transition zone. The coarse aggregate was 5~10 mm continuously graded crushed stone (apparent density 2750 kg/m^3^), and the content of needle and flake aggregate was controlled below 10%. The water-reducing agent is a lignosulfonate condensed polymer high-efficiency water-reducing agent produced by Shandong Wanshan Chemical Co., Ltd. (Jinan, China), with a water reduction rate of 8%. The material system design met the control requirements of GB/T 50080-2016 for high-performance concrete materials [19].

**Figure 1:**
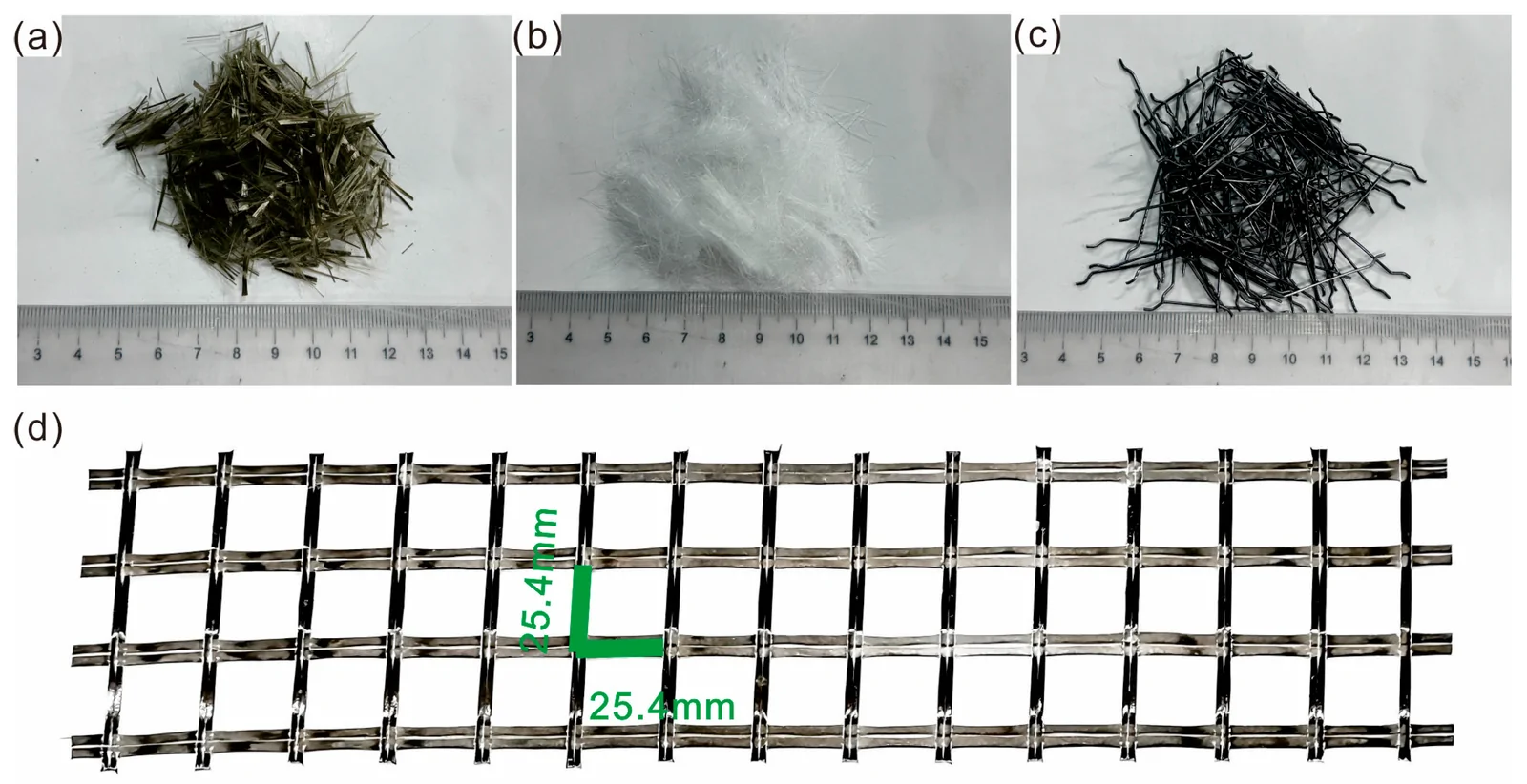
Experimental materials. (**a**) Basalt fiber; (**b**) polypropylene fiber; (**c**) end-hooked steel fiber; (**d**) basalt fiber textile.

We used the BG9090 basalt fiber textile produced by Shandong Junteng New Materials Co., Ltd. (Linyi, China), with a mesh size of 25.4 mm (Figure 1). Specific performance parameters are listed in Table 1 [20]. Basalt fiber produced by Haining Anjie composite materials Co., Ltd. (Haining, China) and polypropylene fiber and end-hooked steel fiber produced by Hengshui Shengying metal products Co., Ltd. (Hengshui, China) (Figure 1) were used for chopped fibers, and their mechanical properties are presented in Table 2.

The above material selection and chopped fibers hybridization were based on the following considerations: 1. chopped basalt fiber and fiber textile share the same composition and thus offer same performance advantages; 2. the end-hooked steel fiber can further enhance the TRC by using its high elastic modulus and tensile strength; and 3. polypropylene fiber can improve toughness through its lower modulus and larger tensile strain. By constructing a modulus gradient combination (basalt polypropylene modulus difference ~100 GPa) and a geometric feature combination (steel-basalt aspect ratio difference of approximately 20 times), the synergistic effect of various chopped fiber hybrids on TRC is explored. After considering factors such as the modification effect of hybrid fiber-reinforced concrete [21,22,23] and the construction feasibility of concrete materials [24], the volume fraction of chopped fibers in this study has been determined as follows: basalt fiber 0.1%, polypropylene fiber 0.1%, and end-hooked steel fiber 0.4%.

### 2.2. Sample Preparation and Test Method

According to the provisions of the CECs 13:2009 standard [25] for test methods of fiber-reinforced concrete, the strength grade of the designed concrete specimens was C40, and the mix proportions of test blocks are shown in Table 3. Thirty bending prisms with the size of 100 mm × 100 mm × 400 mm were fabricated. The control group was plain concrete (group C). The specimens with single chopped fibers included group B (basalt fiber), group P (polypropylene fiber), and group S (end-hooked steel fiber). group N was fiber textile-reinforced concrete. The specimens with additional single fibers on the TRC were BN, PN, and SN groups, corresponding to basalt fibers, polypropylene fibers, and steel fibers, respectively. The BPN and BSN groups mixed two types of fibers, namely basalt-polypropylene and basalt-steel fibers. Each group had three specimens. While conducting prism bending tests, this study conducted cube compression tests on the concrete matrix of each group of samples in accordance with the CECS 13:2009 standard for test methods of fiber-reinforced concrete. The average measured cube compressive strength was as follows: C/N group: 42.09 MPa; B/BN group: 50.89 MPa; P/PN group: 49.42 MPa; S/SN group: 51.73 MPa; BPN group: 44.65 MPa; and BSN group: 49.21 MPa.

**Figure 2:**
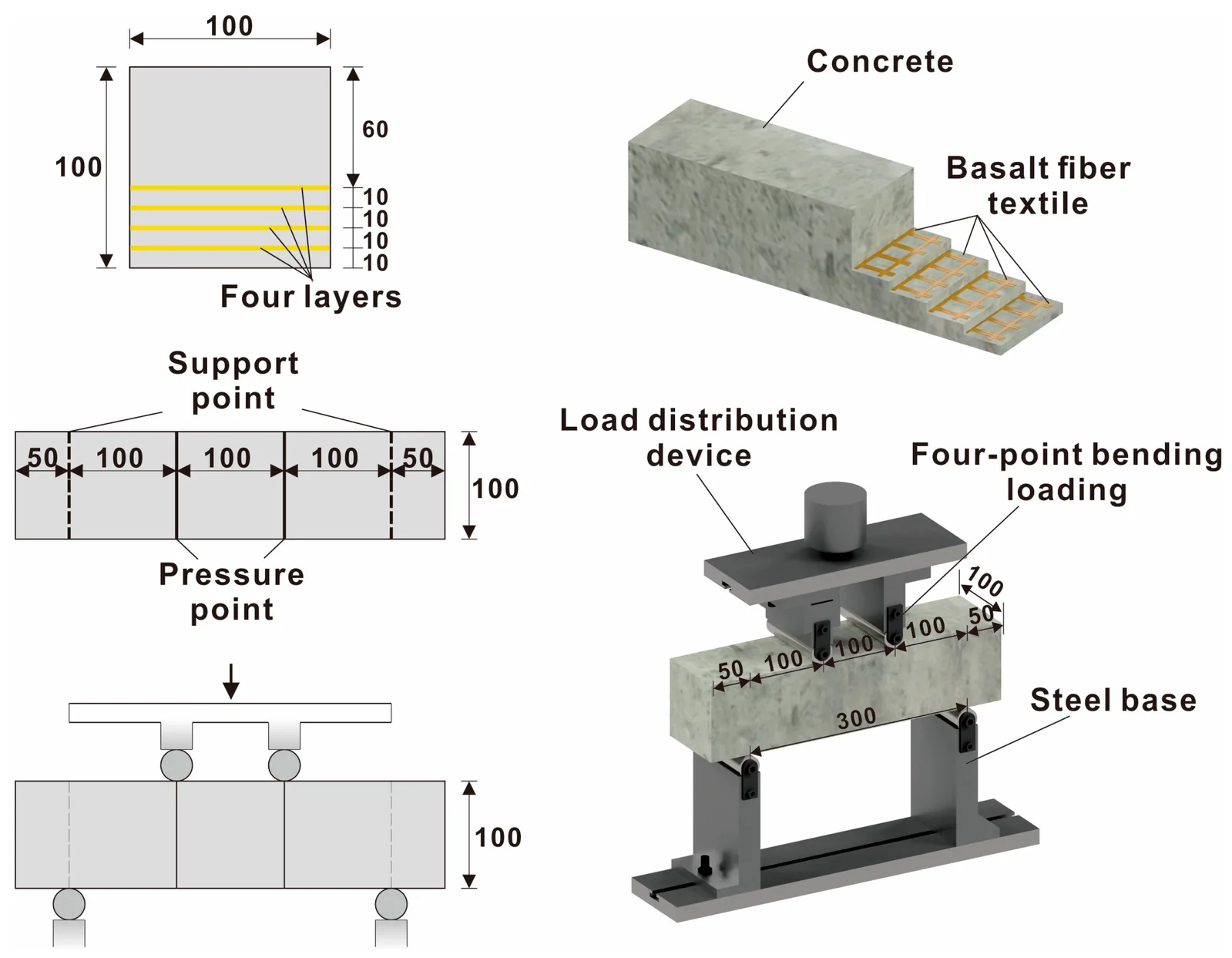
Schematic diagram of specimen size and testing conditions (length unit in mm).

The textile concrete specimens were poured in layers. In this study, four layers of fiber textile were arranged in the specimens. The layers were located 10 mm, 20 mm, 30 mm, and 40 mm away from the bottom of the specimen (Figure 2). The specific procedure was as follows: firstly, 10 mm thick concrete was poured and vibrated and then the basalt fiber textile was laid, then another 10 mm thick concrete was added and vibrated, followed by laying the textile again, and so on to complete the production of four-layer textile concrete specimens.

Chopped fibers were added during concrete mixing. The dispersion characteristics of chopped fibers have an important impact on the performance of concrete. In particular, chopped basalt fibers are easy to fracture, easy to agglomerate, and difficult to disperse. They often gather in concrete to form internal defects, which affect the performance of concrete.

According to the characteristics of chopped basalt fiber, our team explored a mixing process of “secondary pre-mixing and secondary dry mixing” [26]. The steps were to add the dry basalt fiber into the cement twice and to use the powdered cement to stir and disperse the fiber to obtain the dry fiber-premixed cement. Then, sand, stone, and other aggregates were added to the premixed cement and mixed; finally, water reducer and water were added and mixed to obtain concrete mixture. This process ensures good dispersion of basalt fibers in the concrete matrix, low fracture rate, and resistance to agglomeration, resulting in significantly improved performance and good stability of basalt fiber-reinforced concrete compared with plain concrete. Compared with basalt fiber, polypropylene fiber and steel fiber are more easily dispersed. In the process of specimen production, cement, sand, stones, and other aggregates were first mixed, and then polypropylene fiber or steel fiber was added in batches and mixed evenly. Finally, water reducer and water were added and mixed.

After all specimens were formed, they were left at room temperature for 24 h, then the molds were removed and the specimens were numbered. After 28 days of curing in a standard curing room, a four-point bending test was conducted. The test results are shown in Figure 2, and the displacement load sensor accuracy is 0.05%. A continuous and stable loading method was adopted. The initial loading speed was 0.05 mm/min, and when the deflection reached 1.0 mm, the loading speed was adjusted to 1.0 mm/min. The computer automatically collected data and obtained the full load deflection curve.

## 3. Experimental Results and Discussion

### 3.1. Specimen Failure Modes

#### 3.1.1. Chopped Fiber-Reinforced Concrete

**Figure 3:**
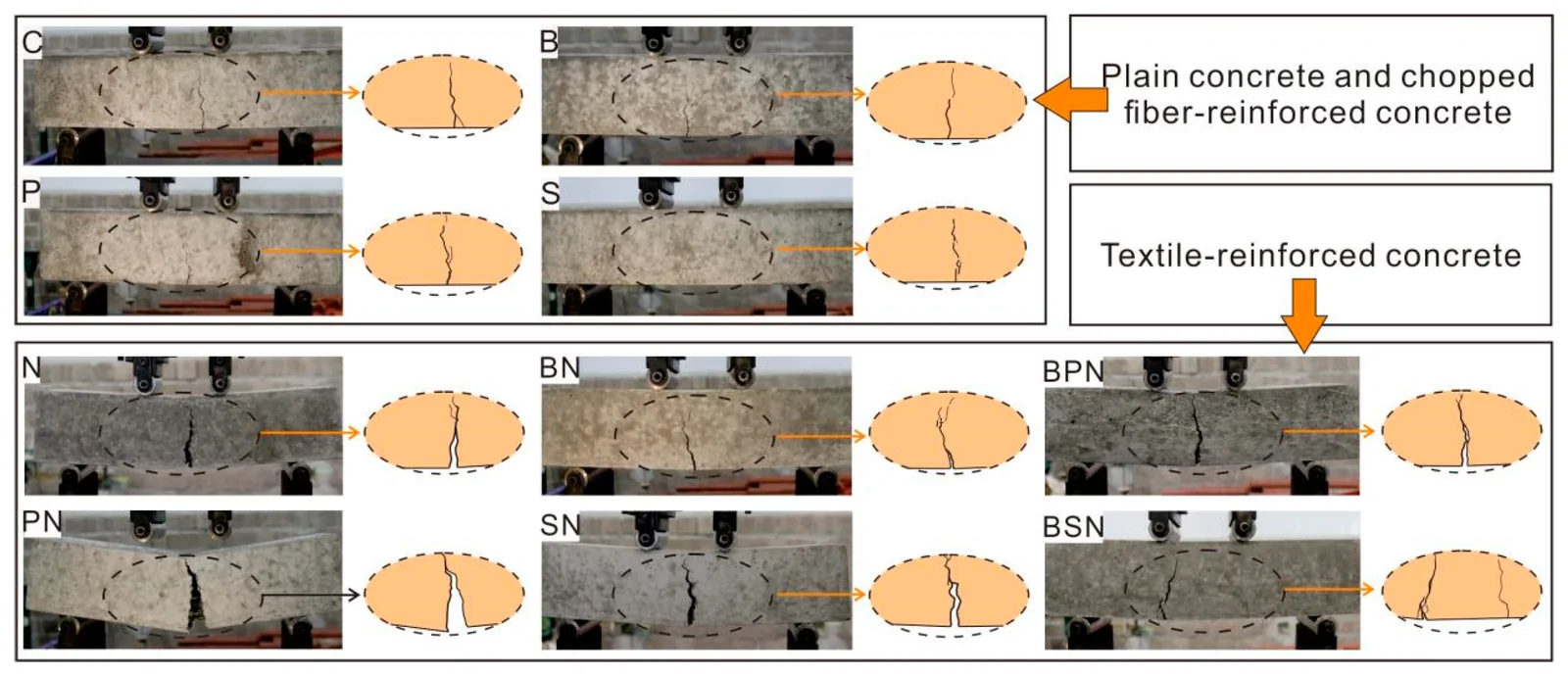
Specimen failure patterns.

The group C, B, and P specimens all exhibited similar failure characteristics: cracks first appeared at the bottom of the specimens, and then they rapidly propagated upward to about two-thirds of the section height, ultimately forming a single main crack that ran through the entire section, with a flat crack surface that exhibited a typical brittle failure mode. In contrast, the failure process of group S exhibited different mechanical behaviors. In the initial tensile stage, an initial crack with a length of about 10 mm appeared in the middle area of the bottom of the specimen. As the load gradually increased, the width of the main crack gradually increased, and multiple branch cracks were derived along the diagonal direction. During the process of crack propagation, steel fibers were continuously pulled out of the matrix, effectively delaying the penetration of cracks and ultimately forming uneven fracture sections, which demonstrated higher toughness and energy absorption capacity. The typical failure modes of each specimen are shown in Figure 3.

#### 3.1.2. Fiber Textile-Reinforced Concrete

During the bending process of TRC specimens, as the load increased, vertical cracks first appeared at the bottom of the specimen, accompanied by a slight tearing sound from the mesh. The cracks quickly expanded to about 10 mm, and then the expansion speed slowed down, significantly owing to the restraining effect of the fiber textile. At different heights where the textile was arranged, the crack paths showed zigzag deviations in various directions.

The failure processes of group BN and group PN were similar to that of group N, all showing a crack development mode mainly controlled by the fiber textile. In contrast, group SN showed slower crack propagation and more significant crack curvature during the loading process, demonstrating better crack control capability. The hybrid effects of multiple fibers showed distinct differences between the BPN and BSN specimens: the failure mode of BPN specimens was similar to that of group N, exhibiting brittle failure characteristics. In the initial stage of loading, the BSN group specimens formed two main cracks at the bottom of the specimens. As the load increased, the paths of the main cracks changed multiple times, and multiple secondary cracks developed. The propagation speed of the main cracks slowed down and their width significantly decreased, showing significant ductile failure characteristics overall (Figure 3).

### 3.2. Flexural Test Results

#### 3.2.1. Initial Crack Load and Peak Load

The flexural test results of the specimens are shown in Table 4. The initial crack load (*F*_cr_) was determined according to the Chinese standard CECS 13:2009 “Test Method Standard for Fiber Reinforced Concrete” [25]. The analysis of the standard deviation and coefficient of variation of the experimental results (Table 5) shows that the coefficient of variation of the initial crack load and ultimate load in most groups is less than 10%, with groups C, P, and N being less than 3.5%, indicating good repeatability of load testing and reliable control of matrix strength and test conditions.

**Figure 4:**
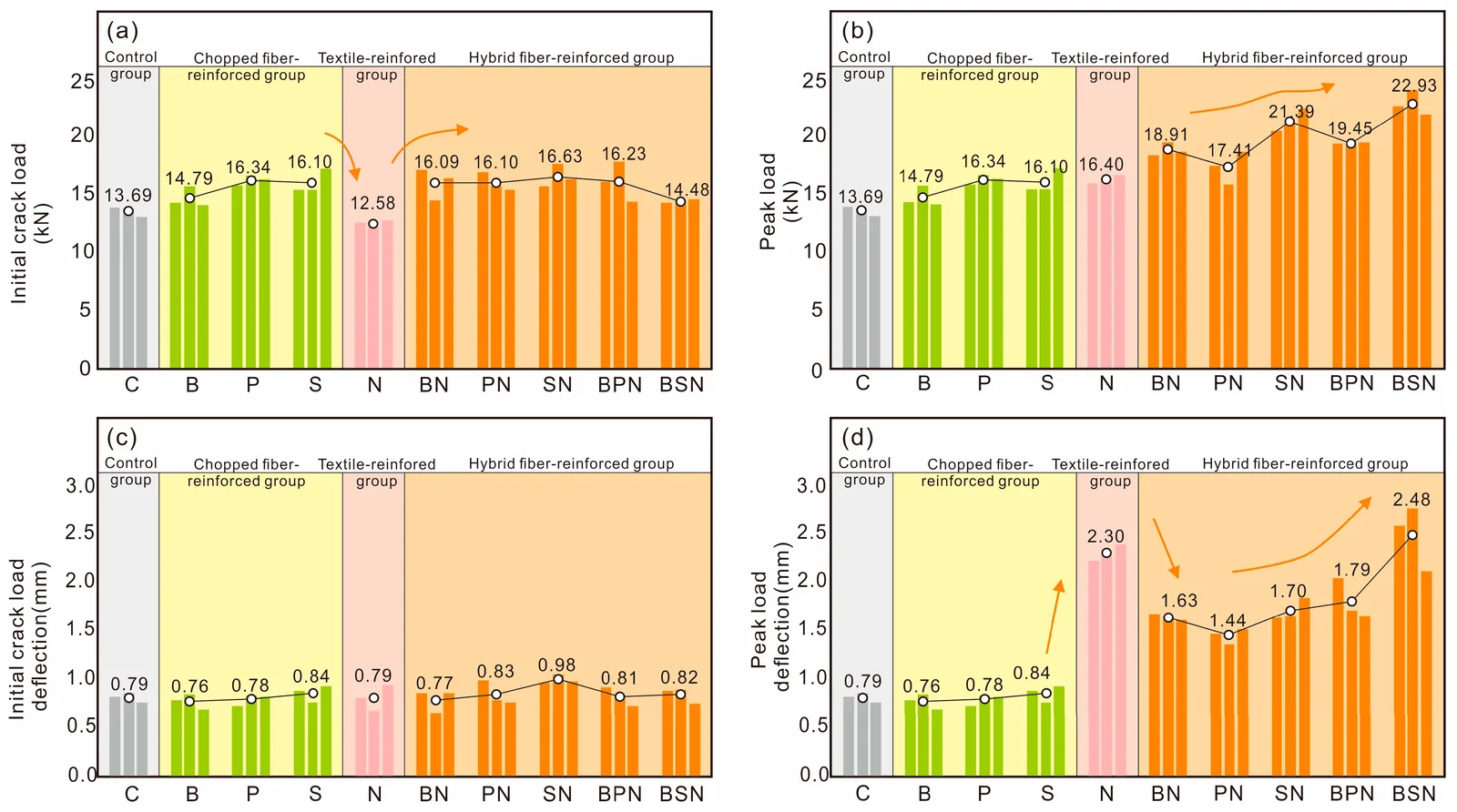
Comparison of (**a**) initial cracking load, (**b**) peak load, (**c**) deflection at initial cracking, and (**d**) deflection at peak load of specimens (all values shown are averages of three specimens).

The results showed (Figure 4) that the average initial crack load of the specimens increased from 13.69 kN of plain concrete to 14.79 kN (an increase of 8%), 16.34 kN (an increase of 19%), and 16.10 kN (an increase of 17%) after adding chopped basalt fibers, polypropylene fibers, and end-hooked steel fibers. Chopped fibers had a restraining effect on crack propagation before crack formation, thereby enhancing the initial crack strength of the specimens. When comparing the results of initial cracking load and peak load, the initial cracking load of the control group specimens was equal to their peak load, indicating that once the bearing capacity of the specimens was lost due to initial cracking, the addition of chopped fibers did not change this phenomenon.

The initial crack load and peak load of TRC specimens exhibited significantly different characteristics (Figure 4). The average initial crack load of group N was 12.58 kN, which was slightly lower than that of plain concrete (13.69 kN). The combined effect of chopped fibers increased the initial crack load of textile-reinforced concrete specimens to 16.09 kN (group BN), 16.10 kN (group PN), and 16.63 kN (group SN), further proving that chopped fibers had a significant effect on increasing the initial crack load of the specimens. Although the fiber textile did not increase the initial crack load of the specimens, the textile-reinforced specimen did not immediately lose its bearing capacity after the initial crack appeared; instead, the load value could rise again and exceed the initial crack value, ultimately reaching a higher peak load. This phenomenon indicates that basalt fiber textile can effectively control the further expansion of cracks after the specimen cracks, thereby improving the ultimate bearing capacity of the specimen.

The peak load showed differences when different types of chopped fibers were added (Figure 4). The peak loads of groups BN, PN, and SN were 18.91 kN, 17.41 kN, and 21.39 kN, respectively, which were significantly higher than those of the TRC specimens without chopped fibers. Among them, the end-hooked steel fibers and fiber textile exhibited the best excellent synergistic effect. The BPN and BSN groups exhibited different properties when two types of chopped fibers were added to the TRC specimens. The initial crack load and peak load of the BPN group were 16.23 kN and 19.45 kN, respectively, which increased by 29.01% and 22.40% compared with the N group. It could be seen that the mixture of basalt fibers and polypropylene fibers increased the initial and peak loads of TRC specimens, but the increase in initial crack load was comparable with that of the single addition of chopped basalt fibers or polypropylene fibers. In terms of ultimate load, the increases were 2.86% and 11.72%, respectively, compared with the single addition of chopped fibers. The initial crack load and peak load of the BSN group were 14.49 kN and 22.93 kN, respectively. Compared with the N group, the initial crack load of this group increased by 15.10% and the peak load increased by 44.30%.

The above results indicate that chopped fibers mainly enhance the initial cracking performance of concrete, while fiber textile significantly improves the bearing capacity and post-cracking development of specimens. The combination of the two, especially the combination of basalt fibers, end-hooked steel fibers, and fiber textile (group BSN), can achieve a synergistic improvement in initial crack strength and ultimate bearing capacity.

#### 3.2.2. Initial Crack Load Deflection and Peak Load Deflection

The coefficients of variation for the initial cracking deflection were between 5% and 17%, while those for the ultimate deflection were mostly between 2% and 14% (Table 5). This is related to displacement measurement accuracy, specimen installation deviation, and the randomness of crack propagation. Among them, the initial crack load deflection of the SN group reached 0.98 mm, an increase of 24.05% compared with the control group, showing a certain improvement. The range of initial crack deflection variation for the remaining test specimens is relatively small, with most averaging between 0.76 and 0.84 mm.

In terms of peak load deflection, the addition of fiber textile significantly improved the deformation capacity of the specimens. The peak load deflection of group N was 2.18 mm, which was 175.94% higher than that of plain concrete. The addition of chopped fibers reduced the deformation performance of the TRC specimens. The peak load deflection of the BN, PN, SN, and BPN groups were 1.63 mm, 1.44 mm, 1.70 mm, and 1.79 mm, respectively, all lower than that of the N group, while the BSN group further increased to 2.48 mm, once again demonstrating excellent deformation capacity (Figure 4).

#### 3.2.3. Load Deflection Curves

##### Chopped Fiber-Reinforced Concrete Specimens

**Figure 5:**
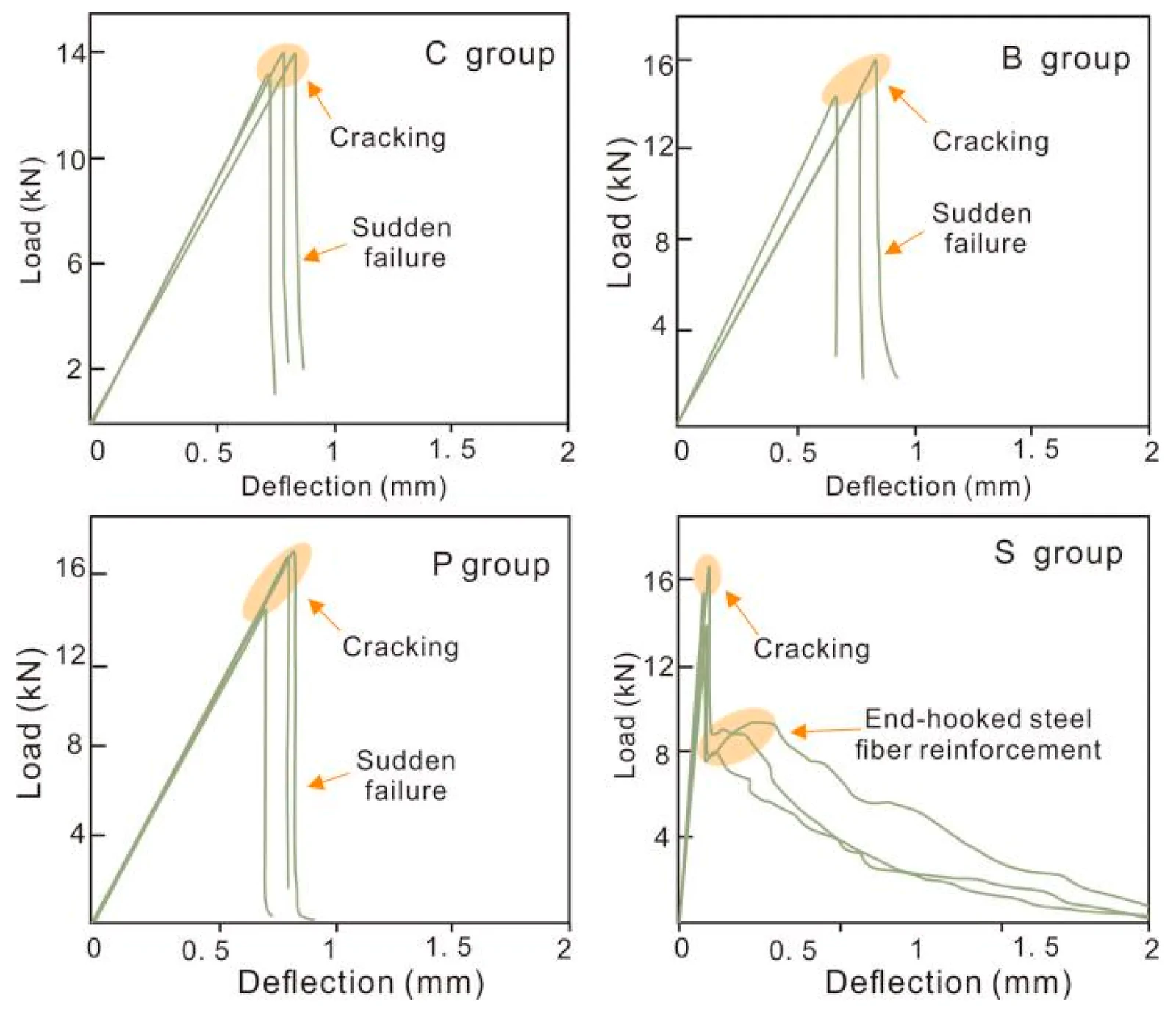
Load–deflection curves of chopped fiber-reinforced concrete specimens.

The load deflection curves of chopped fiber-reinforced concrete specimens (Figure 5) revealed their distinct failure characteristics. All specimens exhibited elastic behavior before reaching peak load. However, their post-peak responses showed significant divergence. The specimens in groups C, B, and P experienced a sharp drop in load after the peak, exhibiting typical brittle failure, consistent with the macroscopic failure process of the specimens. In contrast, group S specimens showed ductile characteristics: the load first decreased to about 50% of the peak load, then briefly rebounded, and then slowly decreased, indicating that the end-hooked steel fibers could still effectively bridge the cracks and maintain the bearing capacity after cracking.

##### Textile-Reinforced Concrete Specimens

**Figure 6:**
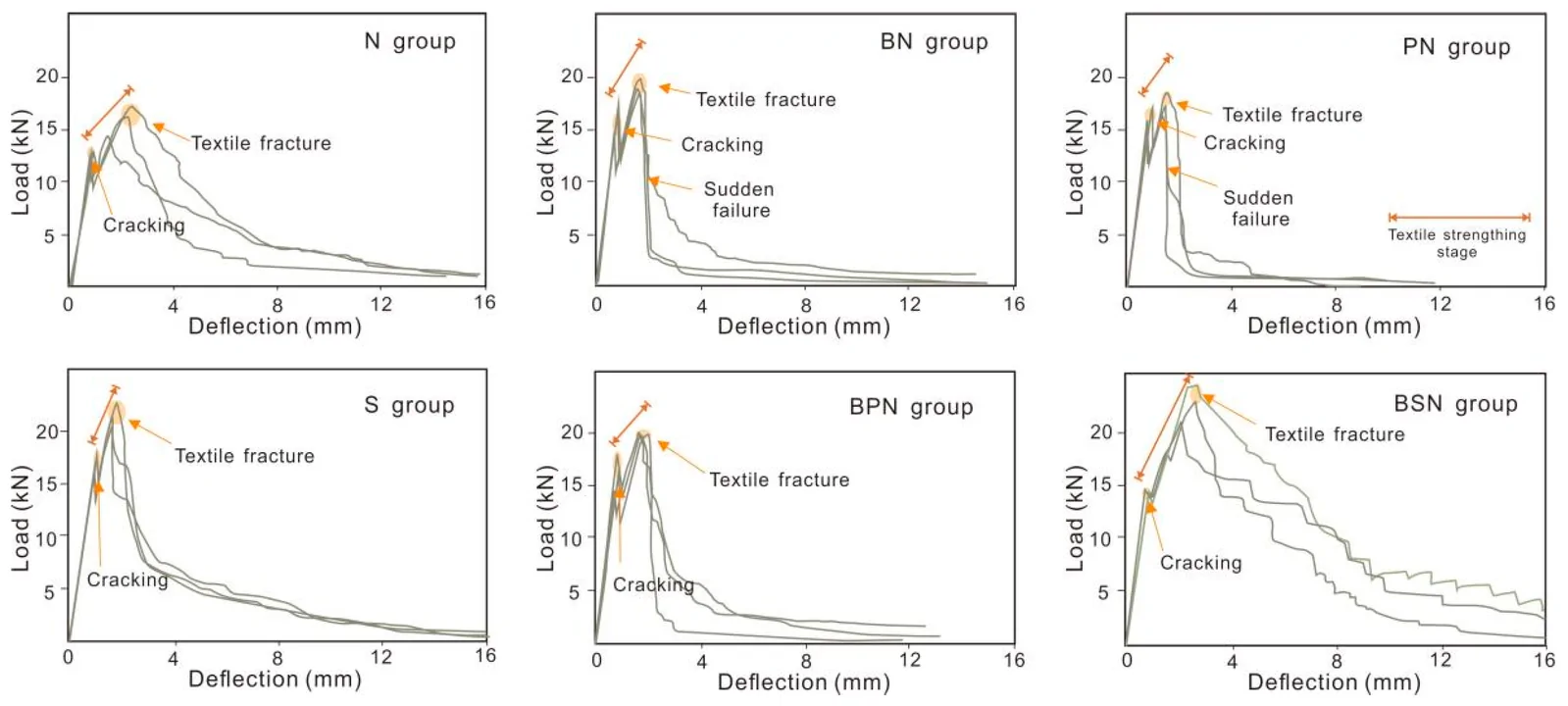
Load–deflection curves of TRC specimens.

The load deflection curves of the TRC specimens showed significant differences compared with those of the chopped fiber-reinforced concrete specimens (Figure 6). For the N group, after cracking, the load did not immediately decrease but entered a fluctuating strengthening stage. This phenomenon is mainly attributed to the collaborative working mechanism between the fiber textile and the concrete matrix: the fiber textile gradually debonded and underwent tensile deformation under tensile stress, resulting in serrated fluctuations in the curves. When the fiber textile began to fracture, the load significantly decreased and the specimen entered the failure stage.

After adding chopped fibers to TRC specimens, different hybrid effects were observed. Compared with the N group, the BN and PN groups had higher peak loads, but their post-peak curves decreased rapidly, showing a trend toward brittle failure. The BPN group also displayed similar characteristics. The addition of end-hooked steel fibers significantly improved the toughness of the system by increasing the peak load and prolonging the wave strengthening stages. Particularly for the BSN group, the post-peak descending slope was notably reduced while the peak load increased, and a residual bearing capacity of over 5 kN was maintained even at deflections greater than 10 mm, showing superior toughness and ductility.

### 3.3. Analysis of Flexural Performance Indicators

According to the Chinese CECS 13:2009 “Test Method Standard for Fiber Reinforced Concrete”, the flexural toughness parameters of the prismatic specimens in this study were determined at the age of 28 days, including the initial crack strength (*f*_cr_), initial crack toughness index (*Ω*_δ_), toughness index at a mid-span deflection of L/150 (*Ω*_k_), flexural toughness indices (*I*_5_, *I*_10_, *I*_20_), equivalent flexural strength (*f*_e_), and flexural toughness ratio (*R*_e_).

The initial cracking strength should be determined according to the following method:

$$f_{\mathrm{cr}}=\frac{F_{\mathrm{cr}}L}{bh^{2}}$$

In the formula:

*F_cr_*—initial cracking load (N);
*L*—the span between the supports of the specimen (300 mm);
*b*—width of specimen (100 mm);
*h*—height of specimen (100 mm).

The calculation method of *f_e_* is as follows:

$$f_{e}=\frac{\Omega_{k}L}{bh^{2}\delta_{k}}$$

In the formula:

*Ω_k_*—the area under the curve for a load deflection of L/150 at the mid-span (N∙mm);
*δ_k_*—the deflection value when the mid-span deflection is L/150 (mm).

The calculation method for the flexural toughness indices f the specimen is as follows:

$$\begin{array}{l} I_{5}=\frac{\Omega_{3\delta }}{\Omega_{\delta }}\\ I_{10}=\frac{\Omega_{5.5\delta }}{\Omega_{\delta }}\\ I_{20}=\frac{\Omega_{10.5\delta }}{\Omega_{\delta }} \end{array}$$

In the formula:

*Ω_δ_*—the area enclosed by the curve at the initial crack deflection value (J);
*Ω*_3*δ*_—The area enclosed by the curve at 3 times the initial crack deflection value (J);
*Ω*_5.5*δ*_—The area enclosed by the curve at 5.5 times the initial crack deflection value (J);
*Ω*_10.5*δ*_—The area enclosed by the curve at 10.5 times the initial crack deflection value (J);
*I*_5_—the flexural toughness index obtained at three times the initial crack deflection value;
*I*_10_—the flexural toughness index obtained at 5.5 times the initial crack deflection value;
*I*_20_—the flexural toughness index obtained at 10.5 times the initial crack deflection value.

The calculation method of flexural toughness ratio is as follows:

$$R_{e}=\frac{f_{e}}{f_{\mathrm{cr}}}$$

**Figure 7:**
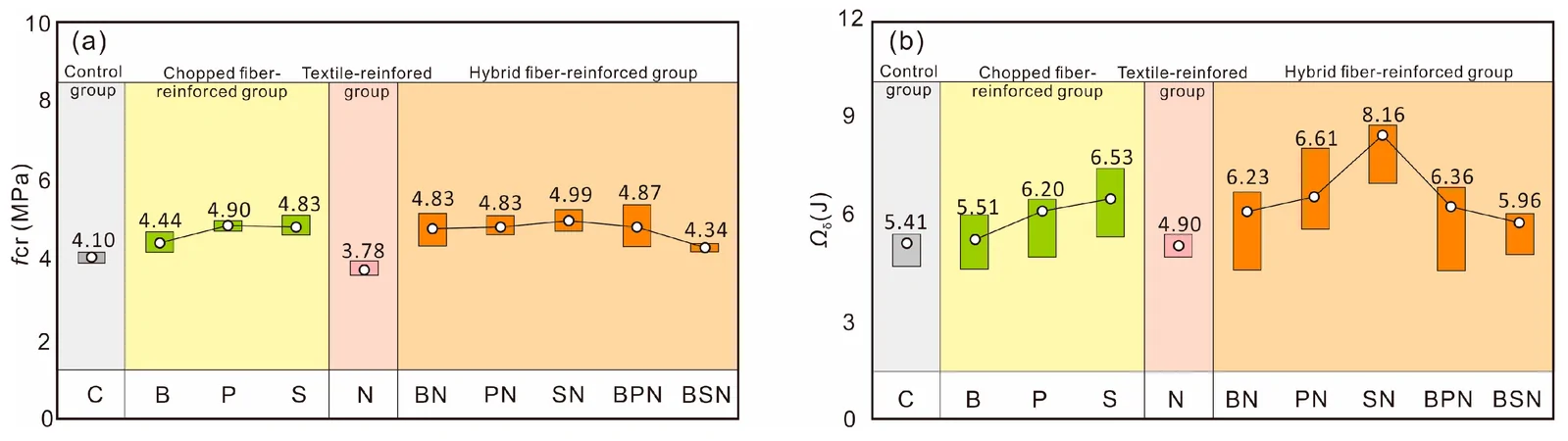
(**a**) Comparison of flexural initial cracking strength (*f*_cr_); (**b**) comparison of energy absorption value at initial cracking deflection (*Ω*_δ_).

According to the initial crack strength (*f*_cr_) results shown in Table 6 and Figure 7a, compared with plain concrete, the specimens with chopped fibers added (groups B, P, and S) showed varying degrees of improvement in *f*_cr_. The initial crack strength of the group N decreased, but further addition of chopped fibers could have compensated for this downward trend.

*Ω*_δ_ is the energy absorption value when the mid-span deflection reaches the deflection corresponding to the initial crack load. As shown in Table 6 and Figure 7b, compared with the control group, the *Ω*_δ_ values of groups B, P, and S all significantly increased, with group S showing the most significant improvement (an increase of 20.7%). The *Ω*_δ_ values of TRC specimens (group N) were slightly lower than those of plain concrete, but when combined with chopped fibers (groups BN, PN, and SN), the initial crack toughness index was significantly enhanced, increasing by 18.48%, 22.18%, and 50.83%, respectively, indicating that the synergistic effect of chopped fibers and textile has a significant effect on improving the initial crack toughness of specimens. However, the initial crack toughness index of the specimens with two types of chopped fibers and textile (groups BPN and BSN) did not show a significant superposition effect.

**Figure 8:**
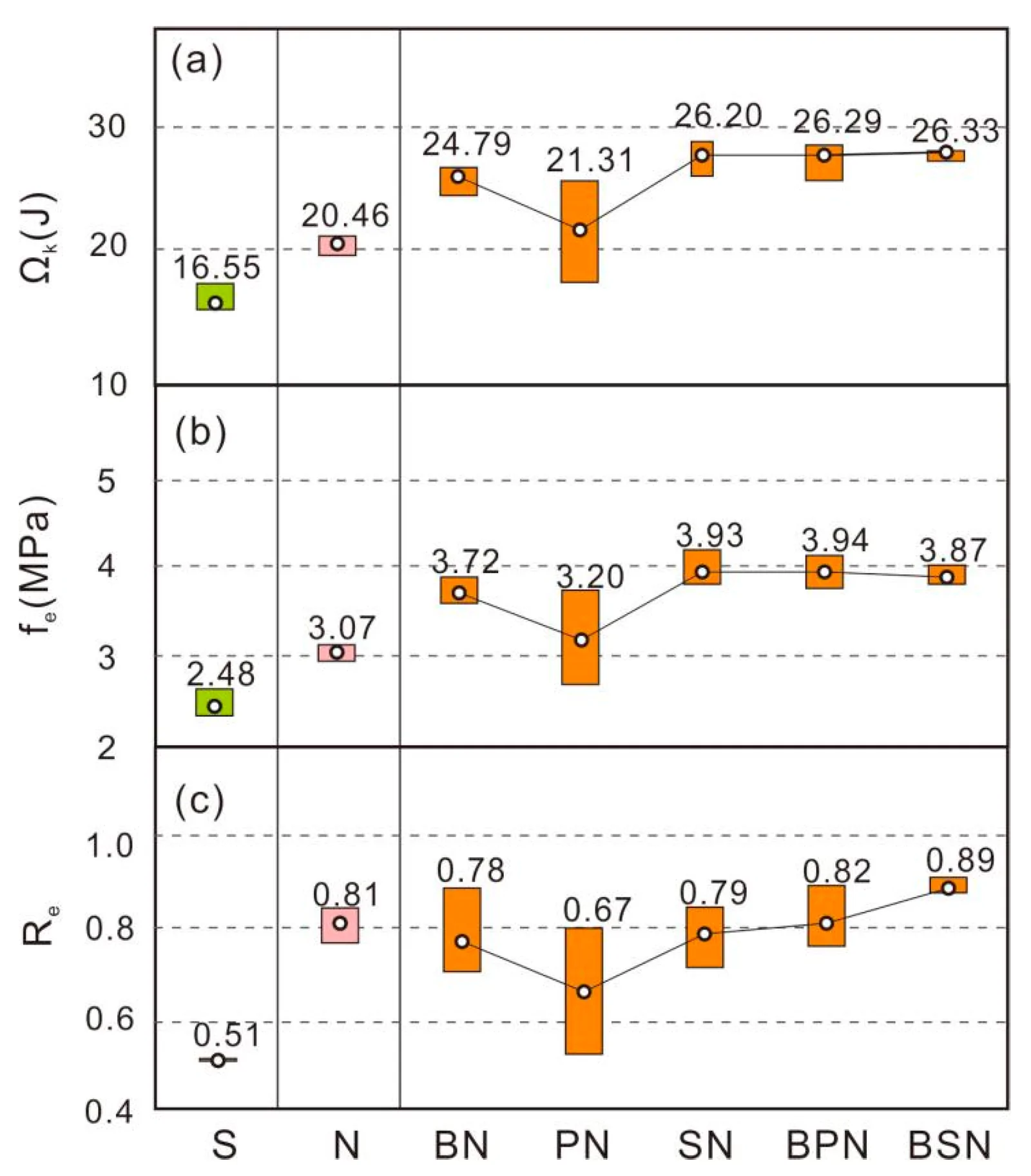
(**a**) Variation curve of energy absorption value (*Ω*_k_) at a deflection of L/150; (**b**) equivalent flexural strength (*f*_e_); (**c**) variation curve of flexural toughness ratio (*R*_e_).

When the deflection reached L/150 or three times the initial crack load deflection, the C, B, and P groups had failed and their toughness parameters (*Ω*_k_, *I*_5_, *I*_10_, *I*_20_, *f*_e_, and *R*_e_) could not be calculated. In contrast, the specimens in groups S and N did not fail under significant deformation, with *Ω*_k_ values of 16.55 J (group S) and 20.46 J (group N), respectively. The combined effect of chopped fibers and fiber textile further increased the *Ω*_k_ values of groups BN, PN, and SN, reaching 24.79 J, 21.31 J, and 26.20 J, respectively. Combining the calculation results of equivalent flexural strength (*f*_e_) and flexural toughness ratio (*R*_e_), it is believed that the BSN group exhibits the best toughening effect (Figure 8b,c).

**Figure 9:**
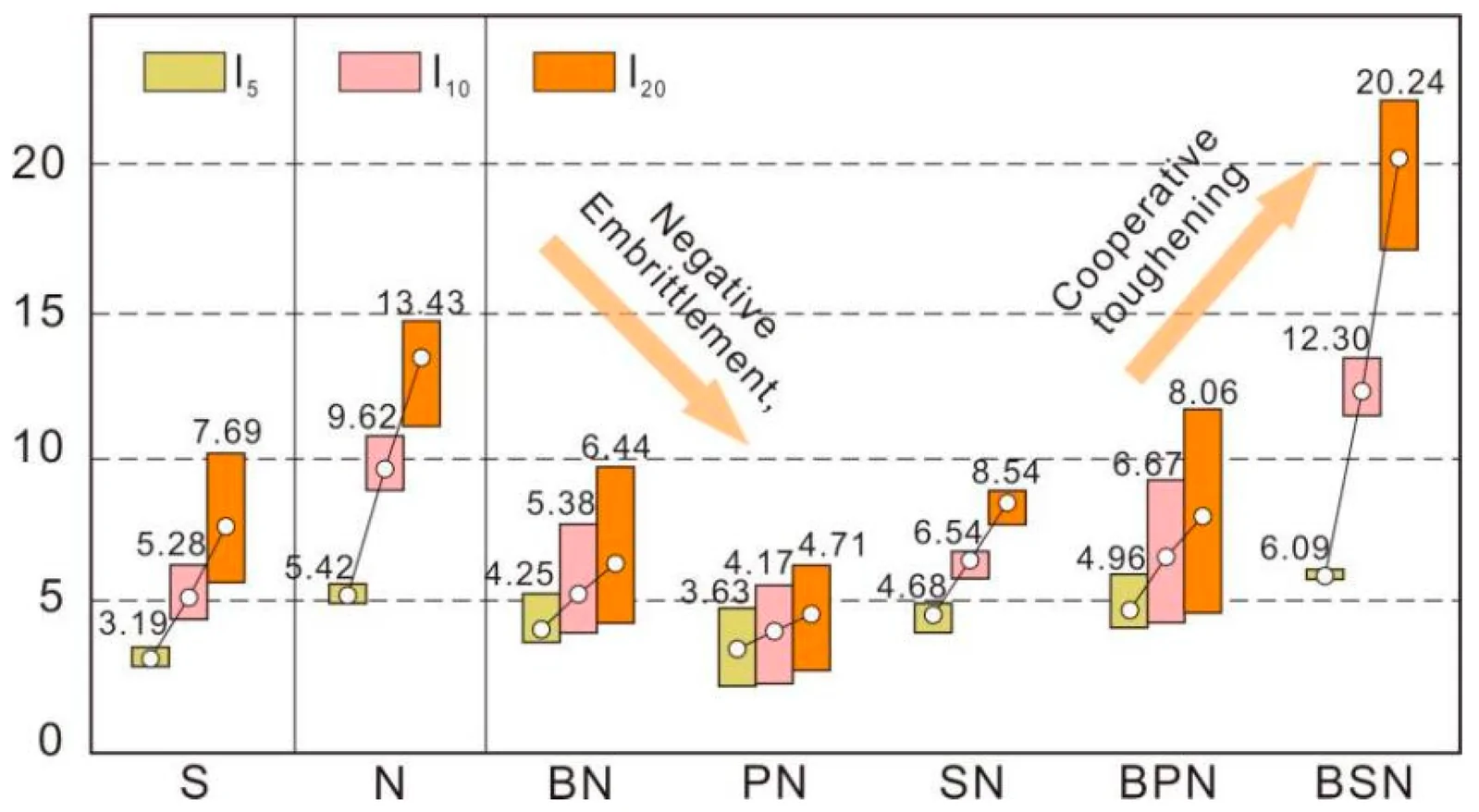
Comparison of flexural toughness indices (*I*_5_, *I*_10_, *I*_20_).

The flexural toughness indices (*I*_5_, *I*_10_, *I*_20_) are used to evaluate the performance retention ability of concrete specimens after cracking. The results showed that compared with group N, the *I*_5_, *I*_10_, and *I*_20_ values of groups BN, PN, SN, and BPN all decreased to varying degrees, showing a negative trend of embrittlement. The *I*_5_, *I*_10_, and *I*_20_ values of the BSN group were 6.09, 12.30, and 20.24, respectively, which increased by 12.36%, 27.86%, and 50.71% compared with the N group, indicating a significant improvement in the post-crack bearing capacity (Figure 9).

Based on the above analysis, it can be concluded that the hybrid effect of chopped basalt fibers, end-hooked steel fibers, and fiber textile can effectively suppress the development of cracks in concrete at different stages of cracking, thereby improving the toughness performance of concrete. The BSN combination is the recommended solution.

### 3.4. Analysis of Fiber Textile Utilization Efficiency

Based on the principle of force equilibrium, the tensile force and utilization rate of the fiber textile under the ultimate state were calculated.

**Figure 10:**
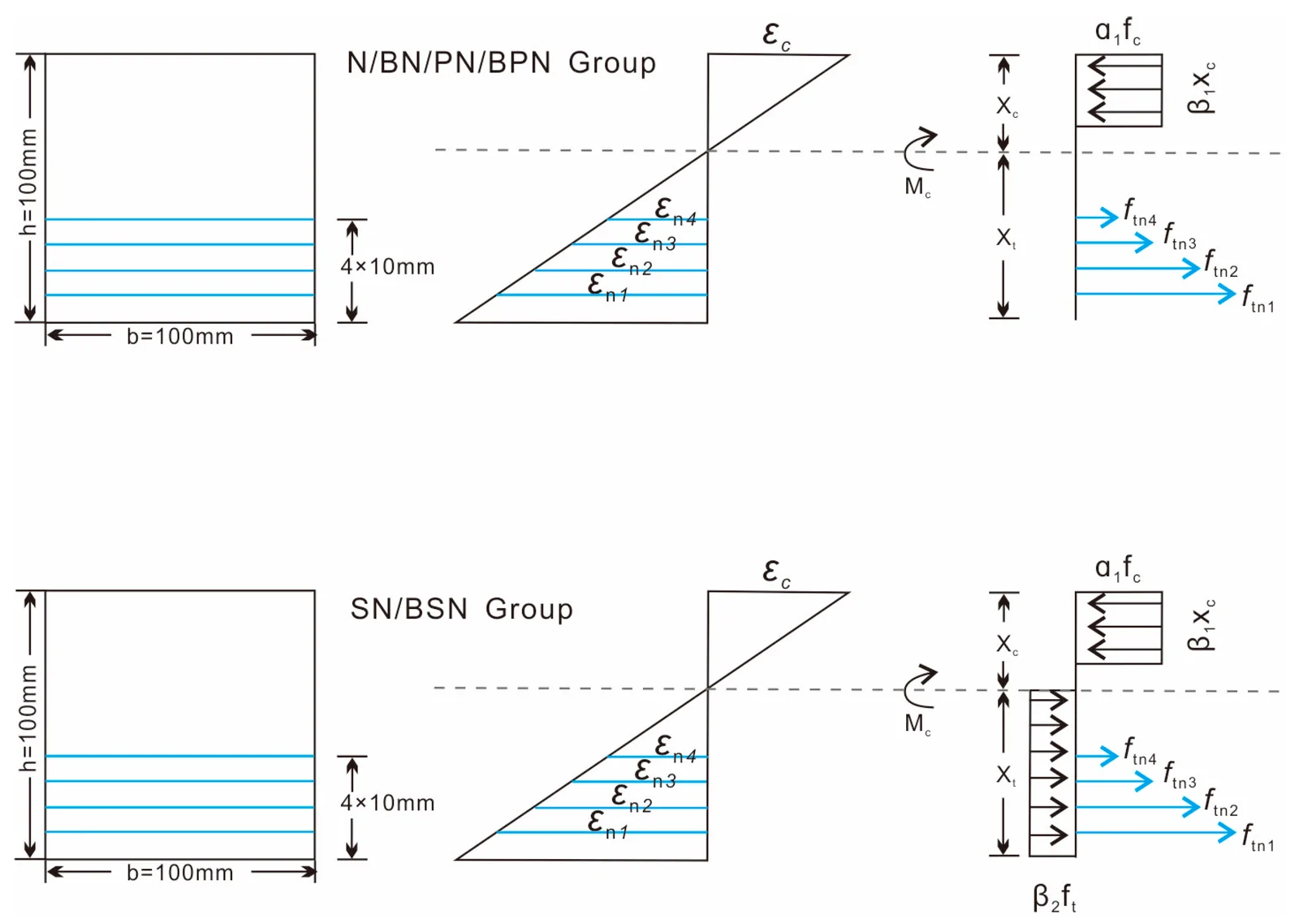
Schematic diagram for normal section calculation of the bearing capacity of specimens, where: b—specimen width (mm), h—specimen height (mm), *X*_c_—height of the compression zone (mm), *X*_t_—height of the tension zone (mm), *f*_tni_—tensile stress of the i-th layer of the textile (MPa), *ε*_i_—tensile strain of the i-th layer of the textile, and *α*_1_ and *β*_1_—coefficients of the equivalent rectangular stress block, taken as 1 and 0.8, respectively.

The tensile strength of plain concrete, basalt fiber, and polypropylene fiber-reinforced concrete is much lower than their compressive strength. In the tensile zone of TRC, the tensile stress developed by the fiber textile is much higher than the tensile stress of the concrete at the ultimate state. Therefore, to simplify the calculation, the tensile force in the tensile zone of the concrete in groups N, BN, PN, and BPN is approximated as the tension of the fiber textile (Figure 10). The tensile strength of end-hooked steel fiber-reinforced concrete is significantly enhanced, and the tensile force is the sum of the tensile force of the basalt fiber textile and that of the steel fiber. To simplify the calculation, the stress curves of the compression and tension zones of the cross-section are taken as equivalent to rectangular stress diagrams.

**Figure 11:**
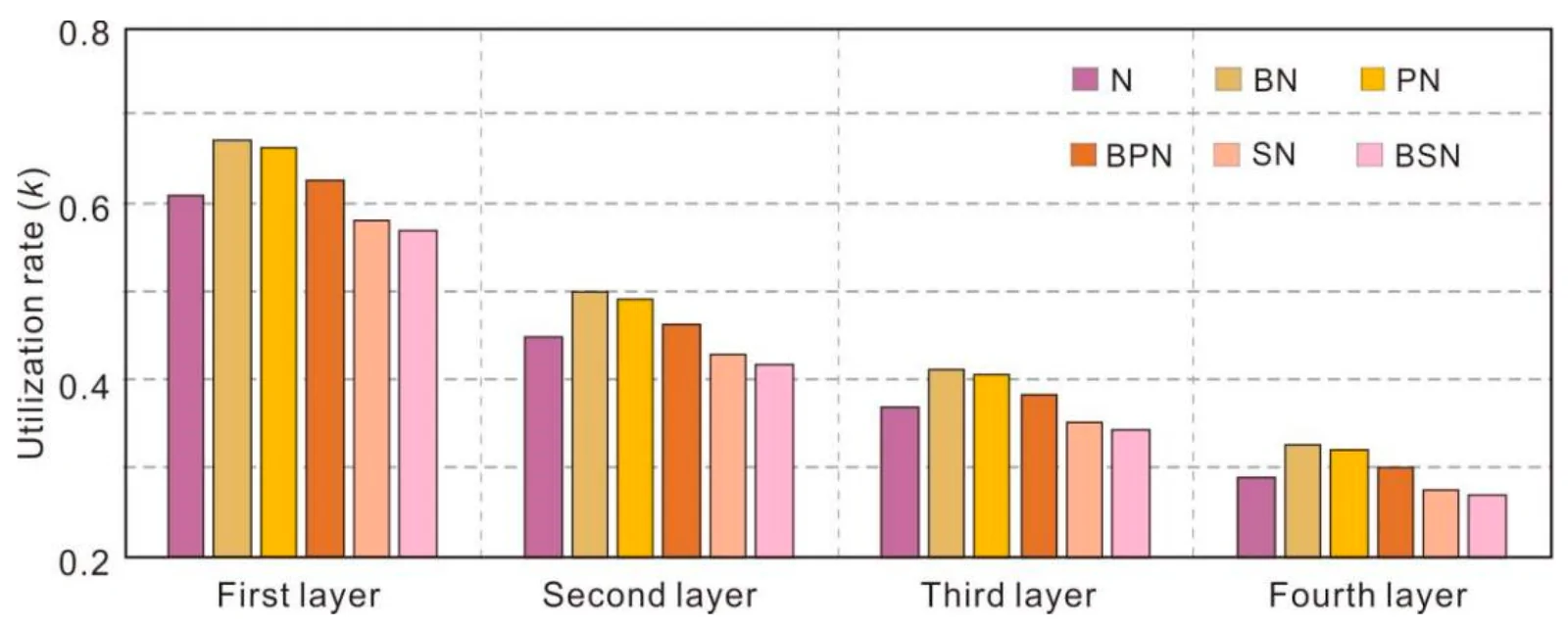
Comparison of the effective utilization rate of fiber textile.

Referring to the calculation method of Hu et al. [20], this paper uses an iterative method to obtain the height of the compression zone (*X*_c_). Based on the assumption of a plane section, the tensile strain and stress of each layer of the textile under the ultimate state were further calculated. The effective utilization rate (*k*_i_) of each layer of the textile is defined as the ratio of the actual tensile stress to its ultimate tensile stress (2340 MPa). The calculation results (Table 7) showed that when the TRC specimen reached the ultimate state, the average utilization rate of the first layer of textile was 0.62. The utilization rates of the second, third, and fourth layers decreased sequentially, with average values of 0.46, 0.38, and 0.30, respectively (Figure 11). Under the action of chopped basalt fibers and polypropylene fibers, the utilization rate of each layer had been improved compared with the N group, with the BN group having the highest utilization rates (0.67, 0.50, 0.42, 0.33, respectively). In contrast, the addition of end-hooked steel fibers significantly reduced the utilization rate of the fiber textile at the ultimate state, and the utilization rates of groups SN and BSN were both significantly lower than that of group N.

## 4. Analysis of Failure Mechanisms

### 4.1. The Influence of Chopped Fibers

Adding chopped fibers to the concrete matrix can inhibit pore connectivity and improve matrix compactness [27], while the bridging effect suppresses the initiation of microcracks and macroscopic crack propagation, improving the mechanical properties of the material [28,29,30,31]. The compressive test results of the concrete matrix in this study showed that the addition of chopped fibers significantly improved the compressive strength of the matrix compared with plain concrete. The prismatic flexural test showed that the initial crack load of specimens in groups B, P, and S was significantly increased by 8%, 19%, and 17% compared with plain concrete, respectively. The flexural initial crack strength (*f*_cr_) and initial crack toughness index (*Ω*_δ_) were also improved to varying degrees, which confirms the improvement effect of chopped fibers.

**Figure 12:**
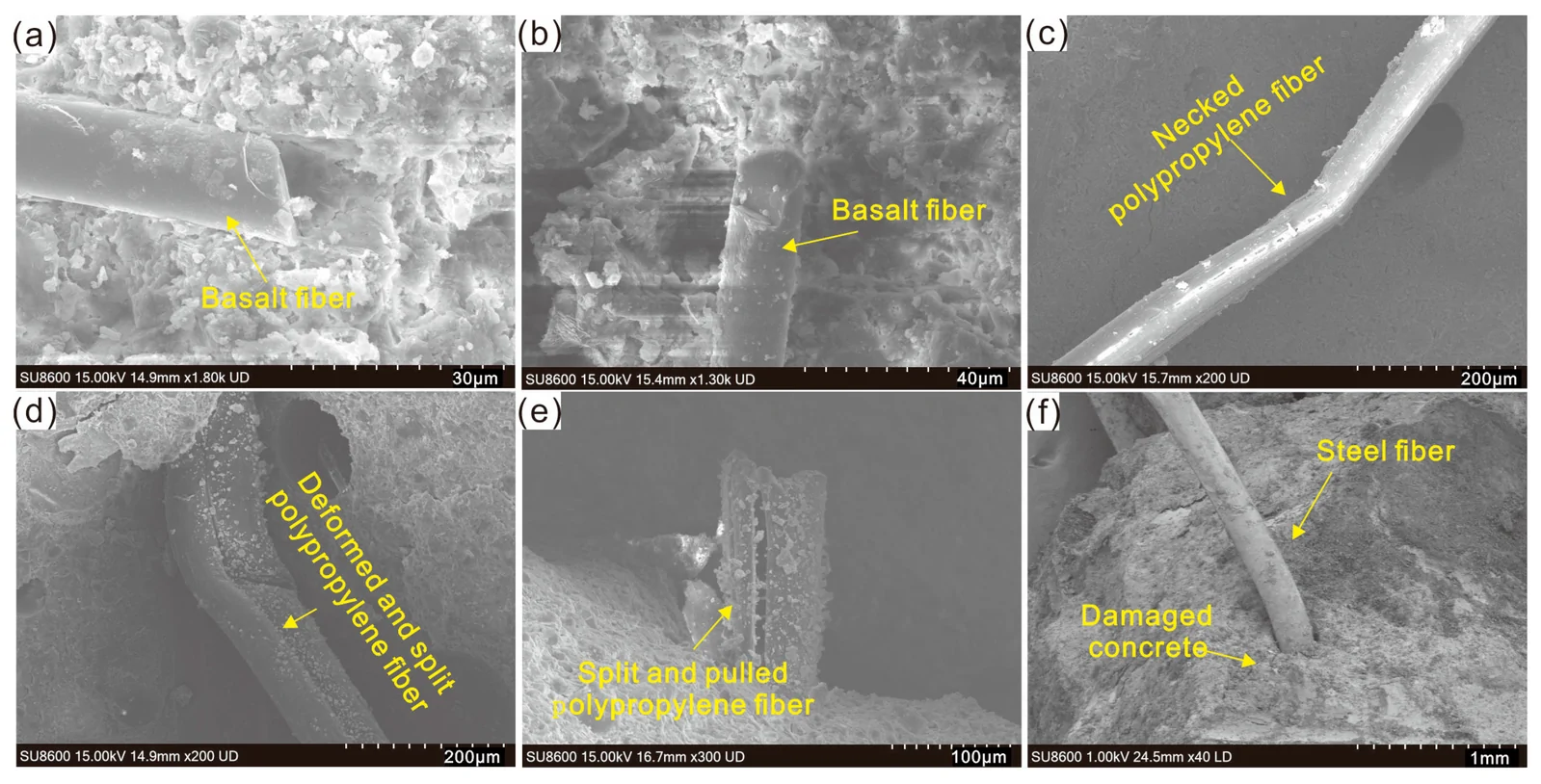
SEM micrographs. (**a**,**b**) Fracture characteristics of chopped basalt fibers; (**c**) Necking characteristics of polypropylene fibers; (**d**) Deformation and splitting characteristics of polypropylene fibers; (**e**) Split polypropylene fibers pulled out from the matrix; (**f**) Pull-out characteristics of steel fibers.

Specimens in groups B and P exhibited brittle failure characteristics, which are mainly attributed to the inherent properties of these two fibers and their interaction mechanism with the concrete matrix. The basalt fiber surface is rich in silicate, which is well bonded with the cement hydration product C-S-H gel [32]. However, their filament diameter is relatively small (7–15 μ m), and although their elastic modulus is high (91–100 GPa), the ultimate tensile strength of the single filament is only 0.2–0.85 N, which is difficult to bear the local tensile stress transmitted by the crack opening after the matrix cracks. Therefore, during the development of macroscopic cracks, basalt fibers are prone to fracture (Figure 12a,b), and their ability to bridge cracks is limited. Therefore, the peak load increase of group B specimens showed only a small increase (about 8%), and it cannot change the brittle nature of the matrix. During the development of cracks in polypropylene fibers specimens, the fibers first undergo debonding, deformation, and shrinkage, accompanied by hardening and splitting along the long axis of the fibers, and then are pulled out of the matrix (Figure 12c–e). This process converts some of the failure energy into the deformation energy of the fibers. As a result, this mechanism increased the peak load by 19%. However, due to the low elastic modulus (3.5~10 GPa) and ultimate tensile strength (300~700 MPa) of polypropylene fibers, they have a high deformation rate and low tensile strength, which make them incapable of effectively inhibiting the expansion of macroscopic cracks. At a polypropylene fiber dosage of 0.1% in this study, the specimens exhibited brittle failure characteristics. Guo et al. also believe that polypropylene fibers have limited toughening effect on concrete under similar dosage conditions (0.12–0.22%) [33]. Nevertheless, polypropylene fibers provide a certain toughness reserve for concrete through the pull-out energy dissipation mechanism, and at higher dosages (0.5–1.5%), polypropylene fibers can improve the ductility of concrete [34]. However, its effect on the workability of concrete must be carefully considered, as high-content polypropylene fibers may significantly reduce the fluidity of concrete [35] and may reduce the strength of the matrix due to agglomeration or increased air content [36,37].

In contrast, the group S specimens exhibited significant ductility characteristics, which are attributed to the unique end-hook anchoring mechanism of steel fibers [38,39,40], and their crack-bridging capacity is positively correlated with the anchoring force of end hooks [41]. The specific process is as follows: the steel fibers first detach from the concrete, and the stress is concentrated in the contact area between the end hook and the concrete [42]. As the stress increased, the concrete in the contact area was locally compressed and damaged, and the end hooks gradually deformed and straightened, ultimately leading to fiber pull-out (Figure 12f). This energy dissipation process endowed the S group with significant ductility, allowing them to absorb energy through fiber pull-out resistance and deformation capacity even after macroscopic cracks appeared, effectively suppressing further expansion of macroscopic cracks. Among the three types of chopped fibers studied, end-hooked steel fibers showed the most outstanding performance in improving concrete ductility due to their anchoring performance.

### 4.2. Influence of Basalt Fiber Textile

During the failure process of TRC, the fiber textile first detaches, deforms, and slides from the matrix until fracture failure [43,44,45], This process effectively controls the development of matrix cracks [46,47].

Test results showed that the initial cracking load of the group N specimens decreased slightly. The initial crack strength and initial crack toughness index also showed decreasing trends. This phenomenon is mainly due to the required cover thickness of the fiber textile and the limitation of coarse aggregate size. The bottom layer of the textile was arranged 10 mm away from the bottom of the specimen, resulting in a lack of effective textile reinforcement in the initial crack development area at the bottom of the specimen, thereby weakening the initial crack resistance of the structure. After cracking, the load on group N did not immediately decrease but entered a fluctuating strengthening phase, and the load deflection curves showed a fluctuating upward trend (Figure 6). This is because as the macroscopic cracks further developed, the fiber textile gradually detached from the matrix and bore the tensile stress. As the tensile strain of the fiber textile increased and the textile began to fracture, the specimens reached their ultimate load, which increased by 19.98% compared with plain concrete.

### 4.3. Hybrid Effect of Chopped Fibers and Fiber Textile

The hybrid effect between chopped fibers and the textile is not a simple superposition of the reinforcement effects of individual materials, but rather it manifests as complex interactions between different fibers and textile [48,49]. This interaction is controlled by the type and properties of the fibers. For example, Zhang et al. found that the synergistic reinforcement effect of steel fibers is significantly better than that of polypropylene fibers, and steel fibers with end hooks are superior to straight steel fibers [3]. The above interactions will affect the performance of concrete from multiple dimensions such as initial cracking performance, ultimate bearing capacity, and post-peak deformation capacity.

#### 4.3.1. Complementary Effect on Initial Cracking Performance

The addition of chopped fibers effectively compensated for the deteriorating trend in the initial cracking performance of fiber textile-reinforced specimens. Specifically, the initial crack loads of the BN, PN, and SN groups were increased to 16.09 kN, 16.10 kN, and 16.63 kN, respectively, and the initial crack toughness indices were improved by 18.48%, 22.18%, and 50.83% compared with plain concrete. These improvements are mainly attributed to the crack inhibiting effect of chopped fibers, particularly in the bottom region where the fiber textile cannot provide effective reinforcement, and where the role of chopped fibers is particularly critical.

#### 4.3.2. Synergistic Effect of Ultimate Bearing Capacity

In terms of peak load, the synergistic effect of chopped fibers and textile has a significant enhancing effect on TRC materials. The peak loads of BN, PN, and SN groups were 18.91 kN, 17.41 kN, and 21.39 kN, respectively, all significantly higher than those of the N group. The peak load of the BSN group specimens reached 22.93 kN, an increase of 44.30% compared with the N group. This indicates that the synergistic effect of chopped fibers can more effectively share external loads and further enhance the ultimate bearing capacity of TRC materials. However, an increase in peak load does not necessarily equate to a positive synergistic effect, and it needs to be judged in conjunction with the synergistic index *SI*. *SI* analysis shows (Table 8) that the BN, SN, and BSN groups have a positive synergistic effect, while the PN and BPN groups exhibit an antagonistic effect.

From a mechanistic perspective, chopped basalt fibers and end-hooked steel fibers enhance the strength of the concrete matrix and may lead to a tighter bond between the concrete and the fiber textile, promoting the release of textile performance. Basalt fibers limit microcracks through bridging effects [50], while end-hooked steel fibers suppress macroscopic cracks [38,39,40]. These complementary reinforcement effects may be the reason for the positive synergistic effect of BN, SN, and BSN groups. On the contrary, polypropylene fibers have a low modulus and undergo debonding and deformation when cracks open, which cannot effectively limit the crack width. This leads to the fiber textile in the crack area bearing greater tensile stress in advance, increasing pre-peak energy release and decreasing post-peak toughness. This may be the main reason for the antagonistic effect observed in the PN group (*SI* = −8.61%). In the BPN group, the positive synergistic effect of BN partially compensates for the negative effect of PN, increasing the *SI* value from −8.61% to −3.47%.

The calculation method of the *SI* value is as follows:

$$\mathrm{SI}=\frac{F_{\mathrm{hybrid},\mathrm{expected}}-F_{\mathrm{hybrid},\mathrm{actual}}}{F_{\mathrm{hybrid},\mathrm{expected}}}\times 100\%$$

*F*_hybrid,actual_—measured average peak load of hybrid system;

*F*_hybrid,expected_—the expected peak load when the short cut fibers are only linearly superimposed on the fabric.

#### 4.3.3. Post-Peak Embrittlement of Deformation Performance

The hybrid effect exhibits a certain complexity in terms of post-peak deformation performance. The addition of chopped fibers significantly reduced the peak load deflection of the specimens compared with group N, and the slope of the load deflection curve became steeper in the descending section. Especially for the PN and BN groups, the post-peak curves decreased rapidly, showing a significant trend toward brittle failure. The evaluation results of the flexural toughness indices (*I*_5_, *I*_10_, *I*_20_) also show the hybrid combination of chopped fibers and the textile exerts a negative embrittlement effect on flexural deformation (Figure 9). This is significantly different from the post-peak toughening effect of steel fibers [13,20], polyvinyl alcohol fibers [41,51], or polyethylene fibers [3] on TRC materials.

This embrittlement effect is related to the effective utilization efficiency of the fiber textile when the specimen reached the peak load. As analyzed above, the utilization efficiency of the fiber textile was improved compared with group N under the action of chopped basalt fibers and polypropylene fibers. More textile stress was released before the specimen reached its peak load, thereby reducing the ability of concrete to maintain its performance after cracking. The negative embrittlement effect of end-hooked steel fibers is weaker than those of basalt fibers and polypropylene fibers. This can be attributed to the bridging ability of end-hooked steel fibers against macroscopic cracks sharing the bending load before and after the cracking, reducing the stress dissipation of the fiber textile, and consequently lowering the textile utilization rate at the ultimate state of the specimens. Ding added macro-polypropylene fibers with a diameter of up to 0.8 mm to the BF-TRC slabs, achieving a post-crack toughening effect [18], which was likewise attributed to the bridging effect of macro-polypropylene fibers on macroscopic cracks.

In this study, group BSN achieved an increase in peak load and exhibited excellent toughness performance, with *I*_5_, *I*_10_, and *I*_20_ values of 6.09, 12.30, and 20.24, respectively, showing improvements compared with the N group, while also having the lowest textile utilization rate (Figure 11). This phenomenon indicates that by reasonably designing a hybrid system of chopped fibers and the fiber textile, the negative embrittlement effect can be alleviated to a certain extent, achieving dual optimization of strength and toughness.

## 5. Conclusions

This study investigated the hybrid reinforcement effect of basalt fiber, polypropylene fiber, end-hooked steel fiber, and basalt fiber textile in concrete through systematic experiments and focused on analyzing their synergistic mechanism. The main conclusions are as follows:

(1) The reinforcement mechanism of chopped fibers exhibits significant differences: basalt fibers and polypropylene fibers can increase the initial cracking load of concrete (by 8% and 19%, respectively), and they cannot change the brittle failure characteristics. The end-hooked steel fibers significantly enhance the toughness of concrete due to their unique mechanical anchoring effect.
(2) Basalt fiber textile improves toughness by controlling macroscopic cracks: fiber textile bears tensile stress together with the matrix, significantly enhancing peak load (increased by 19.80%) and deformation capacity (increased ultimate deflection by 175.94%), reflecting its key role in controlling cracks in the post-crack stage.
(3) The hybrid effect of chopped fibers and fiber textile presents a complex interaction:
  Chopped fibers compensate for the insufficient reinforcement of the fiber textile in the initial cracking area, improving the initial cracking load and toughness (the *Ω*_k_ values of the BN, PN, and SN groups increased by 18.48%, 22.18%, and 50.83%, respectively, compared with the N group).The synergistic effect of chopped fibers and textile can significantly improve the ultimate bearing capacity of TRC, while basalt fibers and end-hooked steel fibers can form a positive synergistic effect with the textile. The mixing of basalt or polypropylene fibers improves the utilization rate of the fiber textile in the ultimate state, which is prone to post-fracture brittleness. In contrast, hybridizing basalt fibers with hooked-end steel fibers can effectively suppress the embrittlement trend by sharing crack loads and delaying the stress release of the textile.
(4) The ternary composite of chopped basalt fibers, end-hooked steel fibers, and basalt fiber textile significantly enhances the toughness performance of cracks at various cracking stages while increasing the peak load, achieving a synergistic improvement in flexural load-bearing capacity and toughness.

This study shows that the hybrid system of chopped fibers and basalt fiber textile can effectively improve the flexural load-bearing capacity and toughness of concrete and has good application prospects in thin-walled components, prefabricated components, and seismic reinforcement engineering. It should be emphasized that in practical applications, the fiber combinations should be designed reasonably, especially to avoid the embrittlement effect that may occur when chopped fibers such as basalt or polypropylene are combined with the textile. It is recommended to choose the synergistic combination of end-hooked steel fibers and basalt fibers and strictly control the fiber dispersion. Future research should focus on interface bonding quality, long-term durability, and construction process optimization to promote the reliable application of this reinforcement system.

## Figures and Tables

**Table 1:** Physical and mechanical properties of basalt fiber textile [20].

	Mesh Spacing/(mm)	Breaking Strength/(kN/m)		Elongation at Break/(%)	
		Warp	Weft	Warp	Weft
Basalt fiber textile	25.4	97	91.2	3.7	3.3

These data were provided by the manufacturer.

**Table 2:** Physical and mechanical properties of chopped fibers.

Fiber Type	Length/(mm)	Diameter /(μm)	Density /(g/cm^3^)	Elastic Modulus/(GPa)	Tensile Strength/(MPa)
Basalt fiber	12	7~15	2.63~2.65	91~110	3000~4800
Polypropylene fiber	12	48	0.90~0.91	3.5~10	300~700
Hooked-end Steel fiber	35	750	7.8~7.9	200~210	800~2000

These data were provided by the manufacturer.

**Table 3:** Test mix proportion (kg/m^3^).

Cement	Sand	Water	Stone (Coarse Aggregate)	Water-Reducing Admixture
425	706	172	1077	1.4

**Table 4:** Initial cracking load, peak load, and corresponding deflection of specimens.

No.	*F_cr_*	*δ_cr_*	*F_max_*	*δ_f_*	No.	*F_cr_*	*δ_cr_*	*F_max_*	***δ_f_***
	kN	mm	kN	mm		kN	mm	kN	**mm**
C-1	13.97	0.8	13.97	0.8	BN-1	17.21	0.84	18.39	1.66
C-2	13.9	0.84	13.9	0.84	BN-2	14.57	0.64	19.56	1.63
C-3	13.2	0.74	13.2	0.74	BN-3	16.48	0.84	18.77	1.6
B-1	14.4	0.77	14.4	0.77	PN-1	17.04	0.97	17.51	1.46
B-2	15.8	0.83	15.8	0.83	PN-2	15.75	0.77	15.94	1.35
B-3	14.17	0.67	14.17	0.67	PN-3	15.5	0.74	18.78	1.51
P-1	15.88	0.71	15.88	0.71	SN-1	15.83	0.95	20.51	1.62
P-2	16.74	0.82	16.74	0.82	SN-2	17.69	1.04	21.19	1.64
P-3	16.41	0.8	16.41	0.8	SN-3	16.38	0.96	22.46	1.83
S-1	15.47	0.86	15.47	0.86	BPN-1	16.19	0.9	19.49	2.03
S-2	15.54	0.75	15.54	0.75	BPN-2	17.96	0.82	19.31	1.7
S-3	17.29	0.91	17.29	0.91	BPN-3	14.53	0.71	19.55	1.64
N-1	12.64	0.79	16.01	2.22	BSN-1	14.4	0.86	22.66	2.58
N-2	12.24	0.66	16.41	2.28	BSN-2	14.4	0.88	24.14	2.76
N-3	12.87	0.93	16.77	2.39	BSN-3	14.64	0.73	21.98	2.11

**Table 5:** Reliability analysis of test results.

**Group**	Standard Deviation				Coefficient of Variation			
	Initial Cracking Load	Initial Cracking Deflection	Peak Load	Peak Load Deflection	Initial Cracking Load	Initial Cracking Deflection	Peak Load	Peak Load Deflection
	kN	mm	kN	mm	/	/	/	/
C	0.43	0.05	0.43	0.05	3.11%	6.34%	3.11%	6.34%
B	0.88	0.08	0.88	0.08	5.97%	10.68%	5.97%	10.68%
P	0.43	0.06	0.43	0.06	2.66%	7.54%	2.66%	7.54%
S	1.03	0.08	1.03	0.08	6.41%	9.74%	6.41%	9.74%
N	0.32	0.14	0.38	0.09	2.53%	17.02%	2.32%	3.75%
BN	1.36	0.12	0.6	0.03	8.47%	14.93%	3.16%	1.84%
PN	0.83	0.13	1.42	0.08	5.13%	15.12%	8.17%	5.68%
SN	0.96	0.05	0.99	0.12	5.75%	5.02%	4.63%	6.83%
BPN	1.72	0.1	0.12	0.21	10.57%	11.78%	0.64%	11.73%
BSN	0.14	0.08	1.1	0.34	0.96%	9.89%	4.82%	13.52%

**Table 6:** Calculated results of flexural toughness indices.

No.	*f_cr_*/Mpa	*Ω_δ_*/J	*Ω_k_*/J	Flexural Toughness Indices			*f*_e_/Mpa	*R_e_*
				*I*_5_	*I*_10_	*I*_20_		
C-1	4.212	5.67	-	-	-	-	-	-
C-2	4.131	5.78	-	-	-	-	-	-
C-3	3.969	4.79	-	-	-	-	-	-
B-1	4.320	5.42	-	-	-	-	-	-
B-2	4.740	6.4	-	-	-	-	-	-
B-3	4.251	4.72	-	-	-	-	-	-
P-1	4.764	6.9	-	-	-	-	-	-
P-2	5.022	5.06	-	-	-	-	-	-
P-3	4.923	6.65	-	-	-	-	-	-
S-1	4.641	6.06	15.9	3.53	6.49	10.29	2.39	0.51
S-2	4.662	5.71	15.98	2.99	4.76	6.85	2.40	0.51
S-3	5.187	7.82	17.77	3.05	4.60	5.93	2.67	0.51
N-1	3.792	4.94	20.91	5.41	8.94	11.14	3.14	0.83
N-2	3.672	5.06	20.72	4.05	7.30	11.48	3.11	0.85
N-3	3.861	5.71	19.76	5.78	10.78	14.76	2.96	0.77
BN-1	5.163	7.15	24.51	3.71	4.12	4.50	3.68	0.71
BN-2	4.371	4.67	25.91	5.36	7.80	9.79	3.89	0.89
BN-3	4.944	6.87	23.96	3.69	4.21	5.02	3.59	0.73
PN-1	5.112	8.51	17.88	2.23	2.47	2.90	2.68	0.52
PN-2	4.725	5.96	21.08	3.81	4.22	4.76	3.16	0.67
PN-3	4.650	5.36	24.98	4.85	5.84	6.48	3.75	0.81
SN-1	4.749	7.42	25.36	5.00	6.89	8.99	3.80	0.80
SN-2	5.307	9.23	25.36	4.13	5.97	7.84	3.80	0.72
SN-3	4.914	7.82	27.87	4.89	6.78	8.79	4.18	0.85
BPN-1	4.857	7.26	25.11	4.16	4.53	4.89	3.77	0.78
BPN-2	5.388	7.18	27.48	4.61	6.13	7.56	4.12	0.77
BPN-3	4.359	4.63	26.29	6.13	9.34	11.72	3.94	0.90
BSN-1	4.32	6.29	25.4	6.04	11.55	17.08	3.81	0.88
BSN-2	4.32	6.45	25.34	6.27	13.44	22.20	3.80	0.88
BSN-3	4.392	5.14	26.71	5.96	11.90	21.43	4.01	0.91

**Table 7:** Calculation results of the utilization rate of basalt fiber textile.

**Group**	*X_c_*/mm	*ε_n_*_1_/mm	*f_tn_*_1_/Mpa	*ε_n_*_2_/mm	*f_tn_*_2_/Mpa	*ε_n_*_3_/mm	*f_tn_*_3_/Mpa	*ε_n_*_4_/mm	*f_tn_*_4_/Mpa	*k*_1_	*k*_2_	*k*_3_	*k*_4_
N	12.86	0.0198	1425.75	0.0147	1056.11	0.0121	871.30	0.0095	686.48	0.61	0.45	0.37	0.29
BN	11.79	0.0219	1575.83	0.0163	1172.85	0.0135	971.36	0.0107	769.86	0.67	0.50	0.42	0.33
PN	11.95	0.0216	1551.86	0.0160	1154.20	0.0133	955.37	0.0105	756.54	0.66	0.49	0.41	0.32
BPN	12.52	0.0204	1470.80	0.0152	1091.15	0.0125	901.33	0.0099	711.51	0.63	0.47	0.39	0.30
SN	13.35	0.0189	1364.32	0.0140	1008.34	0.0115	830.35	0.0091	652.35	0.58	0.43	0.35	0.28
BSN	13.61	0.0185	1333.94	0.0137	984.71	0.0113	810.10	0.0088	635.48	0.57	0.42	0.35	0.27

**Table 8:** Calculation results of the *SI* index.

Group	*F*_hybrid,actual_ /kN	*F*_hybrid,expected_ /kN	*SI*
BN	18.91	17.5	8.06%
PN	17.41	19.05	−8.61%
SN	21.39	18.81	13.72%
BPN	19.45	20.15	−3.47%
BSN	22.93	19.91	15.17%

## Data Availability

The original contributions presented in this study are included in the article material. Further inquiries can be directed to the corresponding author.
